# The P2X_7 _receptor is a candidate product of murine and human lupus susceptibility loci: a hypothesis and comparison of murine allelic products

**DOI:** 10.1186/ar1699

**Published:** 2005-02-21

**Authors:** James I Elliott, John H McVey, Christopher F Higgins

**Affiliations:** 1 MRC Clinical Sciences Centre, Faculty of Medicine, Imperial College, Hammersmith Hospital Campus, London, UK

## Abstract

Systemic lupus erythematosus and its murine equivalent, modelled in the New Zealand Black and New Zealand White (NZB × NZW)F_1 _hybrid strain, are polygenic inflammatory diseases, probably reflecting an autoimmune response to debris from cells undergoing programmed cell death. Several human and murine loci contributing to disease have been defined. The present study asks whether the proinflammatory purinergic receptor P2X_7_, an initiator of a form of programmed cell death known as aponecrosis, is a candidate product of murine and human lupus susceptibility loci. One such locus in (NZB × NZW)F_1 _mice is *lbw3*, which is situated at the distal end of NZW chromosome 5. We first assess whether NZB mice and NZW mice carry distinct alleles of the *P2RX*_7 _gene as expressed by common laboratory strains, which differ in sensitivity to ATP stimulation. We then compare the responses of NZB lymphocytes, NZW lymphocytes and (NZB × NZW)F_1 _lymphocytes to P2X_7 _stimulation. NZB and NZW parental strains express the distinct P2X_7_-L and P2X_7_-P alleles of *P2RX*_7_, respectively, while lymphocytes from these and (NZB × NZW)F_1 _mice differ markedly in their responses to P2X_7 _receptor stimulation. NZB mice and NZW mice express functionally distinct alleles of the proinflammatory receptor, P2X_7_. We show that current mapping suggests that murine and human *P2RX*_7 _receptor genes lie within lupus susceptibility loci *lbw3 *and SLEB4, and we argue that these encode a product with the functional characteristics consistent with a role in lupus. Furthermore, we argue that aponecrosis as induced by P2X_7 _is a cell death mechanism with characteristics that potentially have particular relevance to disease pathogenesis.

## Introduction

Systemic lupus erythematosus (SLE) is a polygenic disease, although the genes contributing towards the disease are unknown. Several human susceptibility loci have been identified, with eight of the strongest candidates mapping to 1q23, 1q25-31, 1q41-42, 2q35-37, 4p16-15.2, 6p11-21, 12q24 and 16q12 [1]. Of the murine models, the New Zealand Black and New Zealand White (NZB × NZW)F_1 _hybrid strain is widely studied due to its similarity to human disease and its female preponderance. As with human SLE, the disorder of (NZB × NZW)F_1 _mice is polygenic with a contribution from both parents. In a study of (NZB × NZW)F_2 _mice, eight susceptibility loci were identified [2]. In the case of the locus *lbw3*, at the distal region of chromosome 5, homozygosity for the NZW-derived locus was associated with increased mortality at 12 months. Although originally mapped to 88 cM on murine chromosome 5 [2], more recent data locate the microsatellite used to define *lbw3 *at 81 cM (discussed later).

We have studied the properties of the proinflammatory purinergic receptor P2X_7_, encoded by a gene within the human SLE locus SLEB4 [3] at 12q24 (Ensembl Genome Browser: ) and by the murine *lbw3 *region (Ensembl Genome Browser: ), and discuss its potential role in disease. The P2X_7 _receptor belongs to a family of ion channels gated by extracellular ATP, but unlike other P2X receptors it is largely restricted to haematopoietic cells. The P2X_7 _receptor has been proposed to play a role in a variety of immune functions including the secretion of leaderless cytokines and the shedding of the lymphocyte homing receptor CD62L [4]. As P2X_7_-deficient mice exhibit resistance to antibody-induced arthritis and impaired CD62L shedding and IL-1β secretion [5], stimulation of this receptor is proinflammatory – suggesting a potential role in autoimmune disease.

In this respect, SLE is of particular interest. Not only is SLE an inflammatory disorder, but it probably reflects, at least in part, an immune response to debris of cells undergoing programmed cell death (PCD). As P2X_7 _stimulation is proinflammatory and induces PCD, functional polymorphisms in this gene would be predicted to affect lupus susceptibility. Moreover, PCD stimulated through the P2X_7 _receptor belongs to a category that bears many of the hallmarks of 'classic' caspase-dependent apoptosis, but also to other categories such as cytoplasmic vacuolization more often associated with necrosis. Such cell death has sometimes been termed 'aponecrosis' [6]. Whereas removal of 'classic' apoptotic cells is believed to be immunologically silent, necrotic cell debris is proinflammatory [7]. The effect of intermediate forms of PCD such as aponecrosis, for which clearance mechanisms have not been defined, is unknown, yet such material potentially plays a significant role in the pathogenesis of SLE (discussed later). Finally, P2X_7 _stimulation results in rapid translocation of phosphatidylserine (PS) from the inner to the outer leaflet of the plasma membrane, which is reversible if stimulation is brief (and thus independent of cell death). As PS and associated proteins are major targets of autoantibodies in SLE [8], cells stimulated via the P2X_7 _receptor may be a significant source of autoantigen in this disease.

An allelic variation (P451L) of the cytoplasmic domain of the P2X_7 _receptor in commonly used mouse strains is associated with significant differences in its sensitivity to the ATP ligand [9]. These allelic forms with proline (P2X_7_-P) and leucine (P2X_7_-L) at position 451 confer high sensitivity and low sensitivity to stimulation by ATP, respectively. While the NZW strain has been shown to express the more responsive allele of P2X_7 _(P2X-P) [9], that expressed by the NZB strain is unknown. We show in the present article that NZB mice and NZW mice express different alleles of the proinflammatory receptor P2X_7_, and furthermore that NZW lymphocytes are markedly more responsive to P2X_7 _stimulation than those from NZB mice. Lymphocytes from (NZB × NZW)F_1 _mice exhibited intermediate sensitivities to P2X_7_-induced PS translocation and to PCD, but were as sensitive to induction of CD62L shedding as those from NZW mice, indicating a comparatively complex phenotypic penetration. The results indicate that P2X_7 _is a strong candidate for being the product of the murine *lbw3 *locus. As the human *P2RX*_7 _gene maps close to SLEB4, we hypothesize that similar polymorphisms may also contribute towards human disease.

## Methods

### Mice

Male mice were purchased from Harlan-Olac (Bicester, UK) and used at between 8 and 14 weeks. Institute guidelines for care of laboratory animals were followed. All studies received ethical review approval.

### P2X_7 _PCRs

PCR amplification of the NZB mouse genomic sequence encompassing the T1352C polymorphism [9] was performed using the forward and reverse primers CCTGTCTAGGCTGTCCCTAT and GCTTATGGAAGAGCTTGGAG for 30 cycles. PCR products were cloned using the TOPO TA cloning system (Invitrogen, Paisley, UK). Forty-three independent clones were sequenced using an ABI PRISM Big Dye terminator ready reaction kit (Applied Biosystems, Warrington, UK) and were analysed on a 3700 DNA Analyser (Applied Biosystems). Nucleotide and amino acid substitutions were numbered using the cDNA sequence accession number NM-011027.

### Reagents

Matrix metalloproteinase inhibitor III was from Calbiochem (Nottingham, UK). Other reagents were from Sigma (Poole, UK), unless stated. Diluents had no effect in any assay used.

### Flow cytometry

Mesenteric lymphocyte cells (10^7^/ml) in phenol red-free DMEM were stained with a combination of CD4^APC^, CD4^CYCHROME^, CD4^PE^, CD8^APC^, CD8^CYCHROME^, CD8^PE^, CD8^FITC ^and CD62L^FITC ^antibodies (Becton Dickinson, Oxford, UK) as indicated, washed and resuspended in DMEM. Cells were equilibrated with annexin V^FITC ^or annexin V^CY5 ^(Becton Dickinson) to assess cell surface PS exposure, or with propidium iodide for 4 min to assess cell death, and were analysed by flow cytometry on a FACScalibur machine using CellQuest (Becton Dickinson) or Flowjo (Tree Star, Ashland, OR, USA) software. Baseline fluorescence was established for approximately 1 min prior to addition of 150 μM (unless otherwise stated) 2'-3'-*O*-(4-benzoylbenzoyl)-adenosine 5'-triphosphate (BzATP). Cells were monitored for PS exposure or CD62L shedding continuously in real time for up to 9 min or were monitored for uptake of propidium iodide, as indicated. All results are representative of at least three independent experiments.

### IL-1β secretion

Spleens from NZW mice and NZB mice were disaggregated and erythrocytes were lysed (Puregene RBC lysis solution; Gentra Ltd, Minneapolis, MN, USA). Splenocytes (5 × 10^6^/ml) were then resuspended in DMEM supplemented with 10% FCS (Helena Biosciences, Sunderland, UK), and were stimulated with 2 μg/ml lipopolysaccharide. After 6 hours at 37°C the medium was removed and replaced with DMEM. Cells were then incubated at 37°C for 30 min with BzATP added as indicated. The supernatant was then collected and the cells and particulate matter were removed by centrifugation. Supernatants were then frozen at -20°C. IL-1β was quantified by ELISA (Quantikine mouse IL-1β kit; R&D Systems, Minneapolis, MN, USA) in accordance with the manufacturer's instructions. Statistical significance was measured by Student's *t *test.

## Results

### Real-time comparison of P2X_7_-stimulated PS translocation and CD62L shedding by NZW, (NZB × NZW)F_1 _and NZB lymphocytes

**Figure 1:**
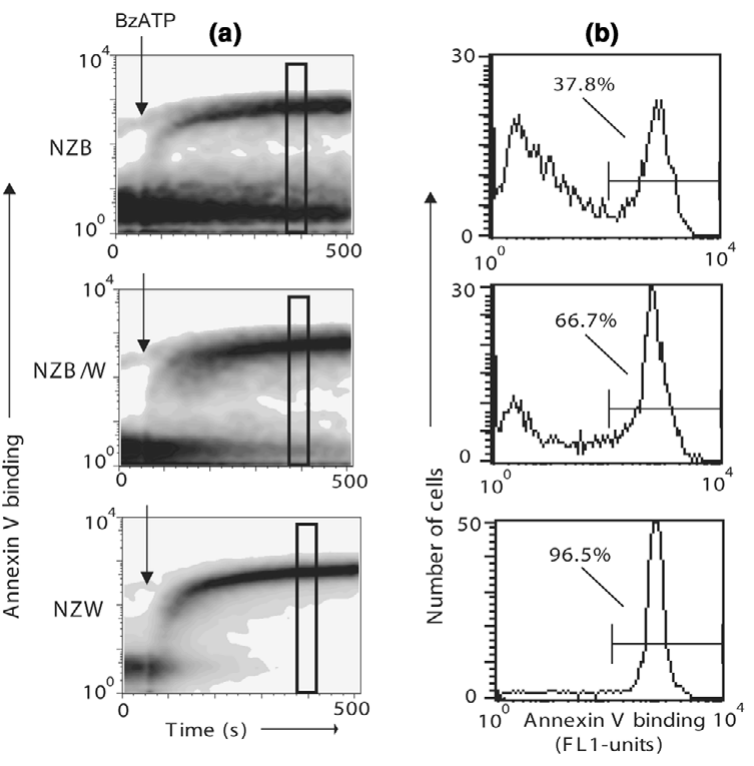
P2X_7_-stimulated phosphatidylserine (PS) exposure on lymphocytes. P2X_7_-dependent exposure of PS on New Zealand Black (NZB) lymphocytes, New Zealand White (NZW) lymphocytes and (NZB × NZW)F_1 _(NZB/W) lymphocytes. To enable the direct comparison of responses of cells from NZB mice, NZW mice and (NZB × NZW)F_1 _mice in a single tube, lymphocytes from these strains were stained with anti-CD4^CYCHROME^, anti-CD4^PE ^and anti-CD4^APC^, respectively, mixed and equilibrated with annexin V^FITC^. Thus labelled, cells could subsequently be distinguished by flow cytometric gating. Cells were stimulated with the P2X_7 _agonist 2'-3'-*O*-(4-benzoylbenzoyl)-adenosine 5'-triphosphate (BzATP) at the time indicated by the arrow in (a). **(a) **Density plots of the rate of extracellular PS exposure in each cell population, as indicated by increased binding of annexin V^FITC^. **(b) **Corresponding percentage of cells bearing exposed PS in each population at a single timepoint (indicated by boxes in (a)).

We initially confirmed that NZW mice are homozygous for the P2X-P allele of *P2RX*_7 _[9] associated with high sensitivity to stimulation, and we showed that NZB mice are homozygous for the low sensitivity allele P2X-L (data not shown). These forms differ at a single amino acid (451). P2X_7 _activation, stimulated by BzATP (Fig. 1), results in rapid externalization of PS and shedding of CD62L. We therefore developed real-time flow cytometric assays to directly compare the responses to P2X_7 _simulation of NZB lymphocytes, NZW lymphocytes and (NZB × NZW)F_1 _lymphocytes.

#### PS translocation

PS is largely confined to the inner leaflet of the plasma membrane in healthy cells. Loss of lipid asymmetry, as evidenced by surface exposure of PS occurring prior to membrane breakdown, is generally assumed to be a marker of PCD. To enable the direct comparison of responses of cells from NZB mice, NZW mice and (NZB × NZW)F_1 _mice in a single tube, lymphocytes from these three strains were stained with anti-CD4^CYCHROME^, anti-CD4^PE ^and anti-CD4^APC^, respectively, mixed and equilibrated with annexin V^FITC ^(to detect exposed PS). Labelled cells could thus subsequently be distinguished by flow cytometric gating. The rates of P2X_7_-stimulated PS exposure by lymphocyte subsets derived from the three mouse strains were directly compared by real-time flow cytometry (Fig. 1). Baseline fluorescence was established, and cells were stimulated with the P2X_7 _agonist BzATP at the time indicated. The order of responsiveness to P2X_7 _stimulation, as evidenced by the percentage of cells translocating PS, was consistently NZW > (NZB × NZW)F_1 _> NZB. Therefore, consistent with the greater sensitivity of P2X_7_-P, lymphocytes from NZW mice show greater sensitivity to P2X_7 _stimulation, and show co-dominance with the NZB-derived allele (P2X_7_-L) in F_1 _hybrid mice.

#### CD62L shedding

**Figure 2:**
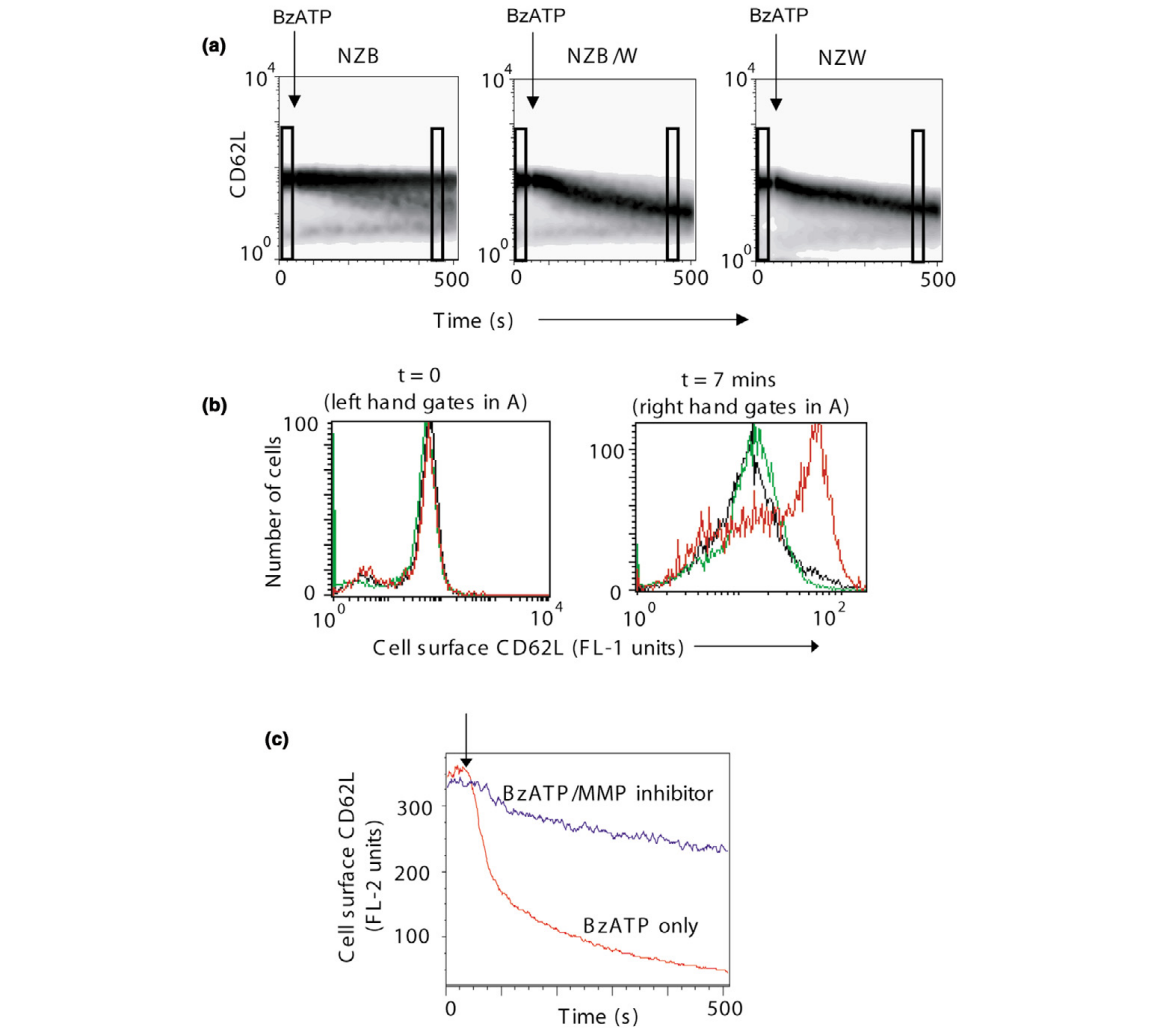
P2X_7_-stimulated shedding of CD62L by lymphocytes. To enable the direct comparison of responses of cells from New Zealand Black (NZB) mice, New Zealand White (NZW) mice and (NZB × NZW)F_1 _(NZB/W) mice in a single tube, lymphocytes from these strains were stained with anti-CD4^PE^, anti-CD4^CYCHROME ^and anti-CD4^APC^, respectively, mixed and stained with anti-CD62L^FITC^. Thus labelled, cells could subsequently be distinguished by flow cytometric gating. Cells were stimulated with the P2X_7 _agonist 2'-3'-*O*-(4-benzoylbenzoyl)-adenosine 5'-triphosphate (BzATP) at the time indicated by the arrow in (a). **(a) **Density plots of the rate of CD62L shedding in each cell population, as indicated by decreased binding of anti-CD62L^FITC^. **(b) **Corresponding levels of cell surface CD62L in each population (NZB, red line; NZW, green line; NZB/W, black line) immediately preceding P2X_7 _stimulation (indicated by left-hand gates in (a)) or 7 min after P2X_7 _stimulation (indicated by right-hand gates in (a)). **(c) **Effect of a broad inhibitor of metalloproteinases on loss of CD62L. Lymphocytes from NZW mice were stained with anti-CD4^CYCHROME ^and anti-CD62L^PE^, and the rate of loss of CD62L was assessed by flow cytometry. Cells were stimulated with BzATP in the presence or absence of 10 μM metalloproteinase inhibitor at the time indicated by an arrow. Shedding of CD62L is indicated by decreased binding of anti-CD62L^PE^. MMP, matrix metallopoteinase.

Shedding of CD62L from T cells is a key event in lymphocyte migration to inflammatory sites [10] and is known to be induced by P2X_7 _stimulation. Lymphocytes from NZB mice, NZW mice and (NZB × NZW)F_1 _mice were differentially stained as already stated but were labelled with FITC-conjugated anti-CD62L in place of annexin V^FITC ^to allow direct comparison of the rate of CD62L shedding in a single tube. P2X_7_-stimulated CD62L shedding was apparent as a decrease in fluorescence in the FL-1 channel. While the rates of P2X_7_-stimulated CD62L shedding were high and low in NZW lymphocytes and NZB lymphocytes, respectively (Fig. 2), interestingly the rate of CD62L shedding by (NZB × NZW)F_1 _lymphocytes was indistinguishable from that by NZW cells. The high NZW response of P2X_7 _thus appears dominant with respect to CD62L shedding, indicating that factors downstream of P2X_7 _stimulation contribute to this phenotype. That loss of CD62L reflects shedding and not decreased cell surface expression through other mechanisms is evidenced by its blockade by an inhibitor of matrix metalloproteinase [11] (Fig. 2c).

### P2X_7_-induced secretion of IL-1β

**Figure 3:**
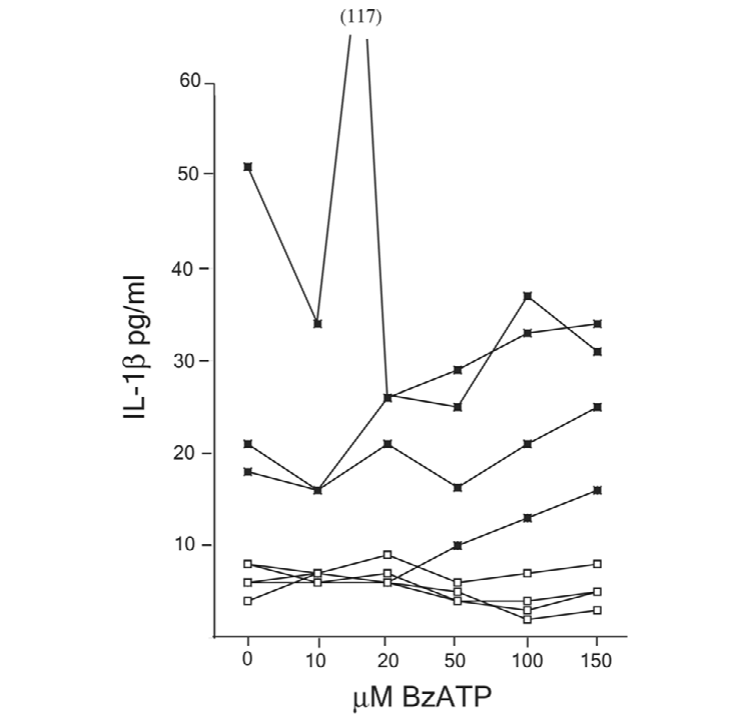
P2X_7_-stimulated secretion of IL-1β. Splenocytes were primed *in vitro *with lipopolysaccharide and were then stimulated with 2'-3'-*O*-(4-benzoylbenzoyl)-adenosine 5'-triphosphate (BzATP) as indicated. The graph shows IL-1β secretion by cells from New Zealand Black mice (open squares) and New Zealand White mice (filled squares). Each line represents IL-1β secretion by cells from a single mouse.

Stimulation of the P2X_7 _receptor on lipopolysaccharide-stimulated monocytes and macrophages promotes secretion of the proinflammatory cytokine IL-1β [5,12], which may therefore be expected to differ between mice bearing P2X_7_-L or P2X_7_-P receptors. Indeed IL-1β secretion by NZW splenocytes stimulated *in vitro *with lipopolysaccharide and BzATP exceeded that by cells from NZB mice (*P *< 0.05 at 50, 100 and 150 μM BzATP; Fig. 3). Elevated IL-1β secretion by NZW splenocytes was apparent even in the absence of BzATP (although slightly below statistical significance), suggesting that inadvertent stimulation of the high (but not low) sensitivity P2X_7 _receptor may have occurred through cell death and the consequent release of ATP during cell preparation (spleen disaggregation and erythrocyte lysis). In one NZW splenocyte preparation exhibiting particularly high IL-1β secretion, cells were refractory to further stimulation of the P2X_7 _receptor *in vitro*.

### P2X_7_-induced PCD of NZW and NZB lymphocytes

**Figure 4:**
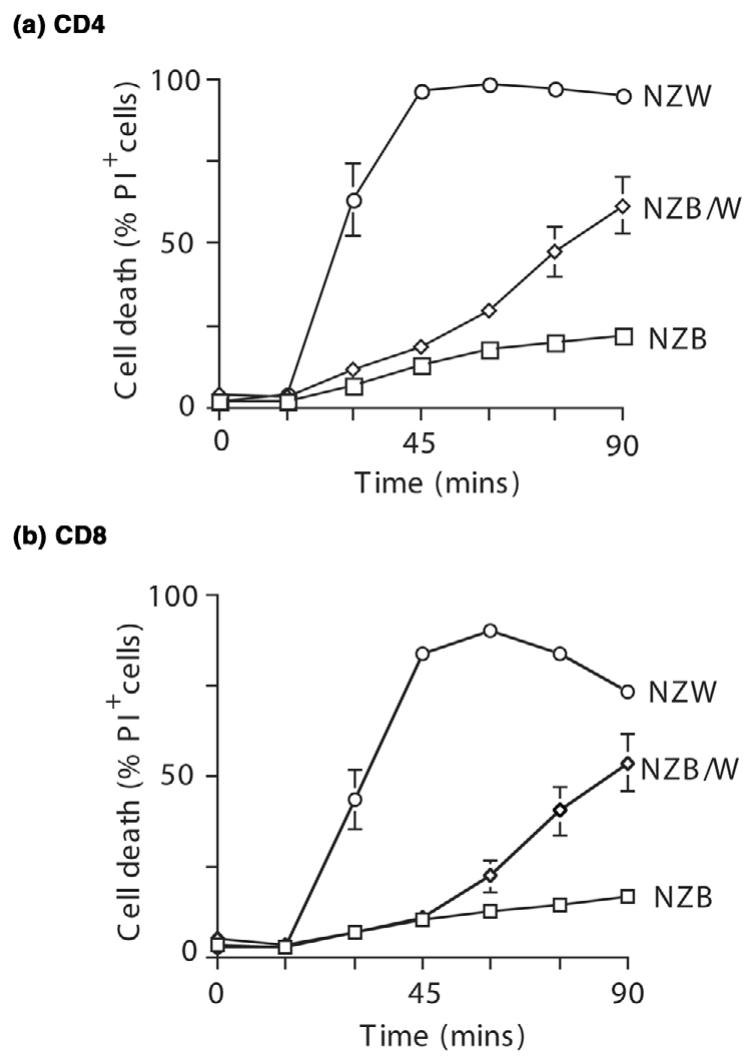
P2X_7_-stimulated lymphocyte programmed cell death (PCD). Lymphocytes from New Zealand Black (NZB) mice, New Zealand White (NZW) mice and (NZB × NZW)F_1 _(NZB/W) mice were stained with anti-CD4^APC ^and anti-CD8^FITC^, and were equilibrated with propidium iodide (PI). Panels show the mean percentage (± standard deviation, *n *= 5) of dead cells (those taking up PI) as assessed by flow cytometry before (*t *= 0), and at 15-min intervals subsequent to, stimulation of the P2X_7 _receptor with 2'-3'-*O*-(4-benzoylbenzoyl)-adenosine 5'-triphosphate. **(a) **CD4^+ ^cells, and **(b) **CD8^+ ^cells. NZB, open squares; NZW, open circles; NZB/W, open diamonds.

Several lines of evidence indicate that lupus reflects an autoimmune response to debris from cells undergoing PCD. Although stimulation of P2X_7 _results in rapid PS translocation, the effects are reversible if exposure to the agonist is brief [4]. Only prolonged treatment with agonist results in PCD. Translocation of PS following P2X_7 _activation cannot therefore be used as a direct measure of irreversible commitment to PCD. To measure PCD following prolonged P2X_7 _stimulation, we therefore compared the rate of terminal membrane breakdown (indicated by propidium iodide uptake) following BzATP treatment of NZW lymphocytes, NZB lymphocytes and (NZB × NZW)F_1 _lymphocytes (Fig. 4). P2X_7 _stimulation resulted in significant PCD in all populations tested, with the order of sensitivity NZW > (NZB × NZW)F_1 _> NZB, consistent with the high responder status of the NZW cells and the dominance of the NZW-derived *P2RX*_7 _allele in this response.

## Discussion

Stimulation of the proinflammatory haematopoietic P2X_7 _receptor [5] results in IL-1β secretion, in high rates of PCD [4] and in CD62L shedding [13], each of which is associated with human SLE [14-16]. The P2X_7 _receptor therefore has the characteristics of a candidate lupus susceptibility gene product. Moreover, the gene encoding human P2X_7 _is located within a region (12q24; Ensembl Genome Browser: ) recently identified and confirmed in Hispanic and European-American Families as a lupus susceptibility locus, designated SLEB4 [3].

A polymorphism in the cytoplasmic domain of the P2X_7 _receptor of common mouse strains is associated with differential responsiveness [9]. While most strains, including NZW mice [9], possess proline in amino acid position 451, we showed that NZB mice express P2X_7 _with lysine at this position and that the variant confers markedly decreased sensitivity to P2X_7 _stimulation. Notably, the murine *P2RX*_7 _gene is encoded by a gene on chromosome 5 within a region designated *lbw3 *due to the identification of a NZW-derived susceptibility locus conferring increased mortality at 12 months [2]. While susceptibility regions are broad, the microsatellite marker *D5Mit101 *(defining *lbw3 *[2]) was in the original study mapped to 88 cM on chromosome 5, which may have discouraged identification of *P2RX*_7 _as a candidate susceptibility gene. Current mapping data, however, show this marker located at 81 cM – Mouse Genome Informatics  and Ensembl Genome Browser . As the marker *D5Mit118 *that is adjacent to *P2RX*_7 _is located at 67 cM – Ensembl Genome Browser  and Mouse Genome Informatics  – the two are approximately 14 cM (or 19 Mb) apart (120 Mb versus 139 Mb), easily within the 20 cM distance used by Kono and colleagues [2] to define coverage by markers in their study.

Although gene polymorphisms may have unpredicted effects, other than *P2RX*_7 _there appear to be few candidate susceptibility genes (based on lymphoid expression and protein activity) in the region described by *lbw3*. However, other candidates might include those encoding: lnk, an adaptor protein in T-cell signalling (65.0 cM) [17]; P2X_4_, a purinergic receptor (65.0 cM) whose activity is assumed primarily to be neuronal, but which is also expressed (at least at the level of mRNA) in lymphocytes [18]; shp2, a tyrosine phosphatase [19] (~66 cM [118.6 Mb]); and FLT3 (CD135, 82.0 cM), a tyrosine kinase expressed in haemaotopoietic cells [20]. None of these has been reported to be polymorphic between NZB mice and NZW mice.

It is widely thought that lupus reflects an autoimmune response to cells undergoing PCD. Aberrant responses to such debris may reflect qualitative or quantitative abnormalities; for example, if its handling is defective and/or following exposure to increased levels of 'apoptotic' material. Both have been reported to contribute to human SLE [15,16]. That prolonged stimulation of the P2X_7 _receptor induces PCD is therefore of particular note. However, multiple pathways of PCD exist. While 'apoptosis' and 'PCD' are frequently used as synonyms, 'apoptosis' is often used to imply caspase-dependent cell death. Nevertheless, caspase involvement is not a good indicator of the physiologic importance, or 'programming', of a cell death pathway, and consequently classic 'apoptosis' may describe one end of a continuum of active PCD mechanisms [21]. Hence, in principle, a defect in one of many PCD pathways, rather than increased susceptibility to PCD *per se*, may be sufficient to increased the burden of cellular debris and hence the susceptibility to lupus. Indeed, that SLE may reflect an autoimmune response to debris from 'apoptotic' cells, despite clearance of such material being thought generally immunologically silent [7], has been a conundrum.

To reconcile these findings it has been suggested that, in SLE, mechanisms for removing apoptotic debris are overloaded, with remaining cells undergoing secondary necrosis, and/or that apoptotic cells have some immunostimulatory properties [7]. We suggest the additional possibility that different forms of PCD may give rise to debris with different degrees of immunogenicity. It is therefore necessary to dissect distinct PCD pathways to assess the potential effects that defects have on the disease process. It is attractive to speculate that P2X_7_-induced aponecrotic debris, perhaps due to the catastrophic nature of its generation or the apparent differences in cell dismantling, may be more necrotic than apoptotic in character and thus be immunostimulatory. Such material may either promote responses to surrounding 'apoptotic' cells and/or directly stimulate autoimmune responses to itself (if lupus autoantigens are appropriately packaged in P2X_7_-induced PCD). P2X_7 _receptor-induced PCD is therefore potentially a source of lupus autoantigens or may represent a catastrophic form of cell death that overwhelms the host's ability to clear such material.

We therefore suggest the following involvement of the P2X_7 _receptor in SLE. ATP exists at very high concentrations in normal cells (5–10 mM), and is released upon cell death before its rapid breakdown by ATPases. Consequently, extracellular concentrations of ATP, although normally low, are transiently increased at sites of tissue damage. Stimulation of P2X_7 _occurs at sufficient concentrations of ATP, resulting in secretion of IL-1β and in CD62L shedding within minutes. P2X_7 _stimulation thus acts to promote the inflammatory response. The resulting lymphoid infiltration leads to additional lymphocyte-mediated cell death, and to consequent ATP release, exacerbating the P2X_7_-driven inflammatory cycle. Indeed, given sufficient tissue damage, prolonged stimulation of P2X_7 _itself induces PCD, further adding to the cycle of ATP release and destruction. Release of autoantigens within P2X_7_-stimulated aponecrotic debris may also contribute to a breakdown in self-tolerance and initiation of autoimmunity.

While one must make the proviso that little is known at the moment about the level of ATP released at sites of tissue damage, its rate of decay and how these may vary between pathological conditions including SLE, we suggest it is reasonable to hypothesize that polymorphisms within P2X_7 _can influence the pathogenesis of lupus. Importantly, there are a number of polymorphisms within P2X_7 _that affect its activity. The Ile-568 to Asn [22], Arg307 to Gln [23], and Glu496 to Ala [24] polymorphisms therefore all result in reduced function of human P2X_7_, and might each be hypothesized to result in decreased severity of SLE.

## Conclusions

In summary, we have shown that polymorphism of the P2X_7 _receptor between NZW and NZB strains is associated with marked differences in P2X_7_-stimulated proinflammatory responses, consistent with high responsiveness and low responsiveness previously reported for the two alleles. We also show that current genetic mapping indicates that the *P2RX*_7 _gene is located within the region defined as *lbw3 *and is a therefore a strong candidate for being the product of this lupus susceptibility locus. Furthermore, as the human gene maps very close to SLEB4, we hypothesize that polymorphisms within *P2RX*_7 _may also contribute to human disease. Stimulation of the P2X_7 _receptor is proinflammatory and induces a form of cell death known as aponecrosis, which exhibits several characteristics of apoptosis. We therefore suggest that the P2X_7 _receptor and gene have the functional and positional characteristics suggestive of a role in the pathogenesis in SLE, and that the potential of the cell death mechanism aponecrosis to contribute to disease warrants study.

## Abbreviations

BzATP = 2'-3'-*O*-(4-benzoylbenzoyl)-adenosine 5'-triphosphate; DMEM = Dulbecco's modified Eagle's medium; ELISA = enzyme-linked immunosorbent assay; FCS = foetal calf serum; FITC = fluorescein isothiocyanate; IL = interleukin; NZB = New Zealand Black; NZW = New Zealand White; PCD = programmed cell death; PCR = polymerase chain reaction; PS = phosphatidylserine; SLE = systemic lupus erythematosus.

## Competing interests

The author(s) declare there are no competing interests.

## Authors' contributions

JIE conceived the study, carried out the flow cytometric and IL-1β secretion experiments, and wrote the first draft of the manuscript. JHM designed allele-specific primers and typed the *P2RX*_7 _genes of NZB mice and NZW mice, and contributed to drafting of the manuscript. CFH contributed to the design of experiments and drafting of the manuscript. All authors read and approved the final manuscript.
